# Universal Model to Support the Quality Improvement of Industrial Products

**DOI:** 10.3390/ma14247872

**Published:** 2021-12-19

**Authors:** Andrzej Pacana, Dominika Siwiec

**Affiliations:** Faculty of Mechanical Engineering and Aeronautics, Rzeszow University of Technology, al. Powstancow Warszawy 12, 35-959 Rzeszow, Poland; d.siwiec@prz.edu.pl

**Keywords:** product quality, nondestructive testing, mechanical engineering, quality management, decision support, quality management tools

## Abstract

Improving the quality of industrial products quality still is a challenge. Despite using quality control, there is a constant need to support this process to achieve an effective, precise, and complex analysis of product quality. The purpose was to develop a universal model that supports improving the quality of products via the consistent and repetitive determination of the causes of product incompatibilities and actions leading to their elimination; the model can be integrated with any quality control of the product. The model verification was carried out for the incompatibility of the mechanical seal in alloy 410, in which the porosity cluster was identified by the fluorescence method (FPI). The purpose of the analysis was created by the SMART(-ER) method. Then, a team of experts was selected from which the brainstorming (BM) was realized. After the BM method, the source of incompatibility and initial causes were identified. Then, the Ishikawa diagram (according to rule 5M + E) was developed to group the initial causes. Next, during the BM method, the main causes were selected. In the last stage, the 5Why method was used to determine improvement actions, i.e., adjust clotting parameters, introduce the obligation to undergo periodic training, and set aside a separate place for storing the electrodes. Originality is the combination of selected quality management tools in a coherent model, the main aim of which is to identify the main causes of incompatibility and improvement actions. Additionally, this model is universal and has applications with analyzing any product and the causes of its incompatibility, and it can be integrated with any product quality control. Therefore, the model can be useful for improving the quality of products in any enterprise.

## 1. Introduction

Achieving adequate product quality includes the necessary actions that allow for repetition of results in industrial conditions [1]. An elementary action in the quality process of achieving the needed quality of products is to identify possible incompatibilities and an effective diagnosis of its source and causes [2]. It refers simultaneously to the need to make an adequate selection of improvement actions [3,4]. Proper stabilization of the production process allows the possibility of organizational development and future development [5,6], and more complex action design and improvement [7,8]. As part of the identified identity of the incompatibility of the product, a popular in industrial practice is the use of nondestructive testing (NDT) [9], which, compared to that of destructive testing (DT), allows control without destroying the product [10]. However, these quality controls allow the identity of incompatibility but do not show the causes of its occurrence [11,12,13]. Therefore, controls of quality are the first stage of improving the quality of product. The analysis of product defects is the process of incompatibility, identifying the causes of occurrence incompatibility [14], which has an impact on the rejection product [11,15,16]. Towards this aim, the key is to use effective instruments. Despite that, after a review of the selected works, it is possible to demonstrate that there is still need for a model that allows for supporting the identification of causes of incompatibility and actions for improvement. The result was mainly due to the ill-considered use of quality management tools. These tools (despite being effective) do not allow for complex analysis of problems when they are improperly selected and used.

According to the literature review, to verify the causes of incompatibility, the Ishikawa diagram is usually employed to identify different possible types of problems or effects. Hitherto, this tool was used to verify the causes of incompatibility of different products, mainly casting [16,17,18,19]; for example, iron-casting defect process [20], metal-casting defect surface [21], or sand-casting [19]. In the last cause, defects were shown to be poorly designed gating systems causing turbulent gating and uncontrolled flow at high temperature of molten metal. Moreover, the Pareto–Lorenz analysis [21] with the Ishikawa diagram was combined. For example, in article [22], the identification of a shrinkage defect in the casting product was performed. The main cause was an inadequate amount of poured metal vertically. In turn, in the work [23], these two tools were combined in the analysis of composite casting defects. Using Pareto, the priority defects of composite steel casting were determined; in turn, the Ishikawa diagram was used to determine the causes of its occurrence. Similar analyses were performed by the authors of the works [20,24,25,26,27], in which the following were analyzed: incompatibility of the iron casting process [25] or causes of occurrence of incompatibilities of the aluminum rims [20], pointing to cramps, inclusions, porosity, and cracks. Furthermore, the analysis of incompatibility causes of casting products (transmission) was presented using the Ishikawa diagram and Pareto analysis in [14]. The main causes of rejection of this product were method errors including filling and solidification, e.g., shrinkage, sand, mold, and water in the casting. A combination of cause-and-effect diagrams with Pareto analysis was also used to analyze incompatibilities in other products than casting, e.g., coil spring plate (CSP) [28], scrapped material [29], metal cover from ABS control unit [30], or automotive products [31]. Additionally, in [14], other popular methods for quality product control were quoted, i.e., brainstorming (BM) and the 5Why method. According to [26], brainstorming (BM) is a teamwork technique, which considers creative thinking and identifies many causes of incompatibility. Relatively often, it was used to generate causes of product defects [11,14,27]. Another method, which was used for quality control, is the 5Why method, which allows identifying the causes of the problem by a fivefold task, the “Why?” question [32].

After reviewing the literature, we found that the most existing publications consider problems with achieving the expected product quality, e.g., [16,18,27]. Frequently, casting products and other products were analyzed [28,29,30,31]. To achieve the effective process of product improvement, it is important to identify the source (place) of incompatibility and causes of its occurrence, and then choose improvement actions. Although various quality techniques were used so far, for example, [22,23,26], mainly the Ishikawa diagram and Pareto analysis, for example, [20,24,25], it was not yet proposed to integrate other quality management tools into a single model, which would be connectable with any quality control process. In this context, this support seems to be legitimate and necessary to achieve an effective, precise, and repetitive analysis of product quality. It is necessary especially in terms of the development of companies, that is, industry 4.0 [33] and the factory of the future. In this area, the need to develop a coherent model was observed, on that will support improving product quality mainly on the stage, make decisions about preliminary (potential) and main causes of incompatibility, and choose adequate improvement actions.

Hence, the purpose was to develop a universal model supporting the improvement of product quality by coherent and repeatable determining causes of incompatibility and actions allowing its elimination, where this model can be integrated with any research of product quality control. This model is named “universal” because all methods selected in the proposed model have universal (general) applications to different kinds of products. Mainly, those are physical products, but also services. The methodologies of the selected methods used in the proposed model have universal applications, where these methods can be used to verify all products and each type of incompatibility. Despite that, in previous studies, these methods were not combined in a single model to complexly verify the quality of the product. As part of achieving the purpose, the hypothesis was assumed:

**Hypothesis:** **1** *It is possible to combine selected quality management tools in a coherent model, the main aim of which is to identify the main causes of incompatibilities and improvement actions, where this model will be universal, i.e., allow to verify any kind of incompatibilities in products detected by any quality control methods.*

The model was tested for incompatibility of the casting product, i.e., porosity cluster on the mechanical seal from alloy 410. These incompatibilities were identified by nondestructive testing (fluorescent method) in the Polish company.

Originality is the combination of selected quality management tools in a coherent model, the main aim of which is to identify the main causes of incompatibility and improvement actions. Additionally, this model is universal and has application to analyze any products and causes of its incompatibility, and it can also be integrated with any product quality control. Therefore, the model can be useful in improving the quality of products in any company.

Section 2 shows the model that integrates several quality control approaches. Section 3 shows the test of this model based on the porosity cluster on mechanical seal from alloy 410. Section 4 is a discussion of benefits, limitations, and future research. Section 5 shows the conclusions of the research.

## 2. Model

### 2.1. Concept of Model

**Figure 1:**
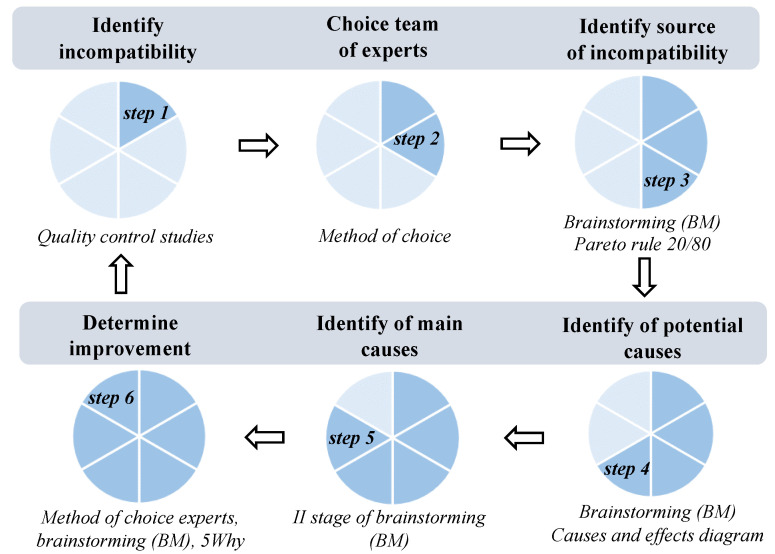
General conception of proposed model.

The universal model to support improve quality of industrial products was developed based on combined quality management tools, i.e., SMART(-ER) method (S—specific, M—measurable, A—achievable, R—relevant or realistic or reward, T—‘based on timeline’ or timebound, E—exciting or evaluated, R—recorded or reward) [34], team of experts of choice [35], brainstorming (BM) [36,37], diagram of causes and effects (diagram of causes and effects (Ishikawa diagram) [16,17,18,19], and the 5Why method [32]. The general concept of the model is shown in Figure 1.

The model has an application to solve problems with incompatibility of products. It concerns identifying the main cause of incompatibility and determining the improvement actions in an adequate way. The concept and simultaneously the main benefits of the model support the determination of causes of incompatibilities and make improvement actions in the area of identifying any kind of incompatibility, which was detected in any product by any method of quality control. Furthermore, the proposed approach specifies the importance of teamwork, which, supported by other additional quality tools, ensures a comprehensive analysis of the problem.

### 2.2. Advantages of Quality Management Tools Conditioning their Combination in the Proposed Model

Based on a literature review, for example, [15,18,24,36,38], the main advantages of selected tools to combine quality management were developed. In the next part, their short characterization is presented.

The SMART(-ER) method [34] has an application to determine the right target, and its choice resulted from these advantages:

- it can be used to determine the aim of the process, influence, results, or personal goals;
- allows determination of actions, participants, interaction, deadline, and effects of actions, knowledge, behavior, and personal preferences.

Brainstorming (BM) [26,36,37]—popular method to generate ideas in the area of analysis, whose choice results in benefits, e.g.,:

- possible to use to generate large numbers of product incompatibility causes;
- possible to apply to generate possible improvement actions in the context of the main causes of product incompatibility.

The Causes and Effects Diagram (or Ishikawa Diagram) [15,38]—the so-called fishbone diagram or herringbone diagram, which allows for the identification and visualization of different kinds of possible causes and occurrence problems and effects. The choice of the Ishikawa diagram resulted in benefits, for example:

- possibility of systematized and uncomplicated determination of potential (initial) causes of incompatibility occurrence;
- possibility of grouping causes according to categories, for example, rule 5M + E, i.e., man, machine, material, management, environment, and measure;
- according to the authors of [39], it allows one to solve 90% of all product quality problems;
- possible to implement this diagram in the stage of initial (potential) causes.

The 5Why method [32]—allows for the identification of the causes of the problem by asking five questions “Why?” until the source of the problem is identified, and it is also applied to determine improvement actions. Therefore, it was adequate to use this method in the proposed model.

In turn, some limitation of the proposed methods is the need to skillfully combine them as part of the analysis of the product incompatibility and determine improvement actions. Otherwise, their effectiveness decreases, and it is possible to wrongly determine the causes of incompatibility. As a consequence, inadequate improvement actions are taken, which causes a waste of resources. Hence, the main assumptions of the proposed model were made, as is shown in the next part of the article.

### 2.3. Assumptions of Model

To develop the model, the main assumptions were made. These results from the specific advantages and limitations of selected quality management tools. These assumptions were:

- lack of limitations in determining the number of incompatibility causes;
- lack of limitations in determining the number of improvement actions;
- recognition incompatibility causes are equally as important;
- need for historical data about incompatibilities and improvement actions;
- the model is realized in a properly selected team of experts;
- possibility of analyzing any incompatibilities detected by any product quality control method.

**Figure 2:**
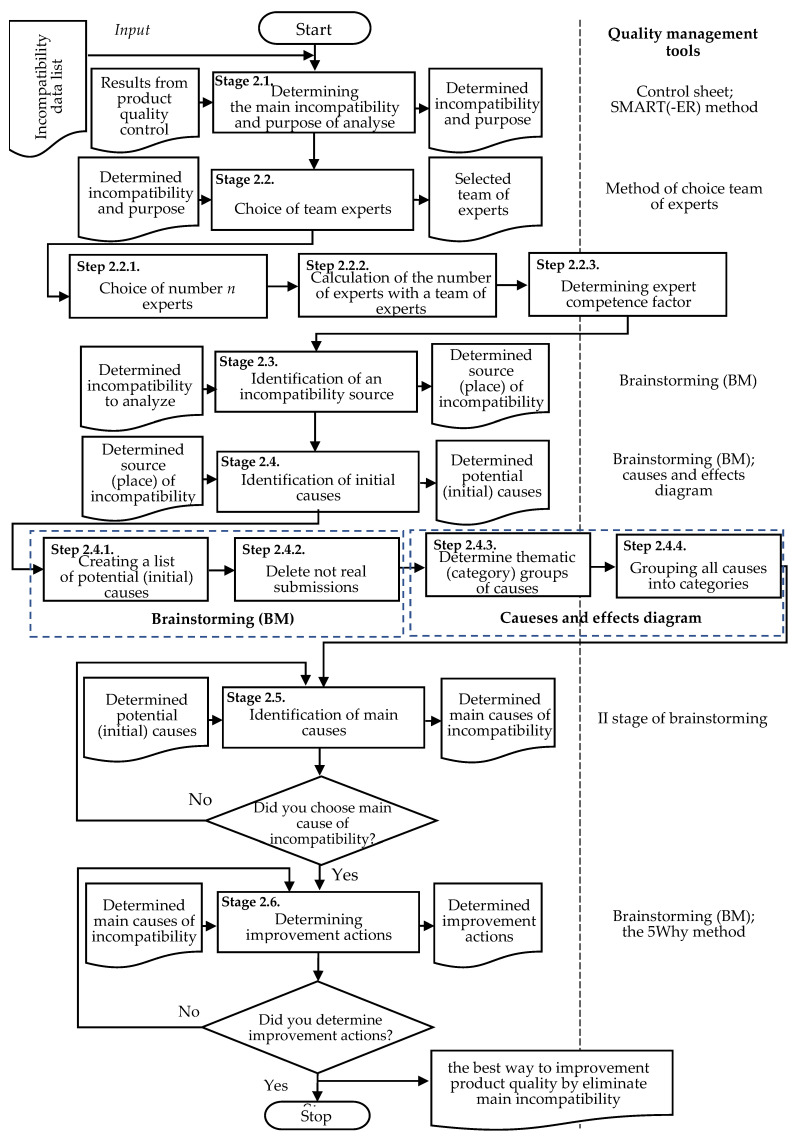
Universal model to support improve product quality integrated with quality management tools.

These assumptions were fundamental to developing a coherent combination of these tools in a single model and then integrate these tools into the process of product quality control. The developed model is presented in Figure 2.

The characteristic of the proposed model is shown in the next part.

- Stage 2.1. Determining the Main Incompatibility and Purpose of Analyzing

The incompatibility with analyze is mainly the main incompatibility, i.e., the most often the occurrence, having the biggest cost or effects. This incompatibility can be selected based on the control sheet realized by the company. According to the main incompatibility it is necessary to assume the aim of analyze. The purpose has to be determined by the entity (company, expert—manager, president et al.). To determine the objective in the right way, it is necessary to use the SMART(-ER) method [34]. The right definition of aim included: kind (name, type) of incompatibility, the kind of product on which this incompatibility was occurrence, and the number of this incompatibility (on an annual basis). This information can be obtained from the catalogue of incompatibility or control sheets.

- Stage 2.2. Choice of Team Experts

The choice of team experts is realized to assign responsibility for carrying out the product quality improvement process (i.e., identification of main causes and proposed improvement actions). The choice of experts should allow a team to be created in which each expert will have knowledge and competences about the problem analysis theme. Towards this aim, it is proposed to apply the method of choice team of experts, which is shown in three steps.

◦ Step 2.2.1. Choice of Number n Experts

The entity of method choice and experts. He is guided by the knowledge of the experience of these experts and these experts will be competencies.

◦ Step 2.2.2. Calculation of the Number of Experts with a Team of Experts

Following the authors work [35], it is assumed that each of the n experts determines the same number of experts in your group (z = n). Hence, the minimum number of experts is determined by Formula (1):

(1) $$N>\frac{n\times z\times \left(n-1\right)}{\left(n\times z-\sum_{i\;=1}^{n}\mu \left(i\right)\right)+1},$$

where: N—required number of experts, z—choice of competent experts by n selected experts, μ(i)—number of unique experts by i-th experts of *n* groups of experts.

If each expert gives the same number of experts, the required number of experts is estimated according to Formula (2) [35]:

(2) $$N=\frac{n^{2}\left(n-1\right)}{\left(n^{2}-\sum_{i\;=1}^{n}\mu \left(i\right)\right)+1},$$

where: N—required number of experts, μ(i)—number of unique experts by i-th experts of n groups of experts.

◦ Step 2.2.3. Determining Expert Competence Factor

To determine the degree of expert competence, the competence factor is used. According to [35], this factor is estimated using Formula (3):

(3) $$\begin{array}{ccc} K_{k}=\frac{k_{z}+k_{a}}{2} & \mathrm{where} & k_{z}{\;\mathrm{and}\;k}_{a}\;∊\;\langle 0;\;1\rangle \end{array},$$

where: K_k_—factor of expert competence, k_z_—factor of degree knowledge of the problem by experts, k_a_—factor of arguments.

The factors of the degree knowledge of the problem by experts (k_z_) and factor of argument (k_a_) are determined by self-assessment by experts. A set of rating scales for the competence factor were used for the assessment (Table 1).

Each expert determines his own knowledge of the problem on a scale from 0 to 10 (according to Table 1). Then, the points determined by the expert are multiplied by the value of 0.1. according to Formula (4) [35]:

(4) $$\begin{array}{ccc} k_{z}=p\times 0.1 & \mathrm{where} & k_{z}\;∊\;\langle 0;\;1\rangle \end{array},$$

where: p—rating awarded by the i-th expert.

On the other hand, the argumentation factor takes into account the so-called structure of arguments, which is the basis for the expert grades. The argument factor is determined by the expert according to Table 1, and as shown, Formula (5) [35]:

(5) $$\begin{array}{ccc} k_{a}=a_{1}+a_{2}+a_{3} & \mathrm{where} & k_{a}\;∊\;\langle 0;\;1\rangle \end{array},$$

where: a_1_, a_2_, a_3_—arguments (as in Table 1).

The higher experience in factor k_a_ is the higher practice experience than the theoretical experience. For example, k_a_ = 1, k_a_ = 0.75, k_a_ = 0.5 appropriately: high–average–low degree of influence of all argumentation sources on the expert’s opinion.

Following the authors [35], it was assumed that the threshold value of the competence factor (K_k_) is equal to 0.6. Therefore, from all experts, it is necessary to remove experts whose factor K_k_ is lower than the value 0.6 (6):

(6) $$T=N\;-\left(N\;∊{\;K}_{k}<0.6\right),$$

where: N—required number of experts, K_k_—factor of expert competence.

The obtained number of experts with T is the number of competence experts to analyze the problem. Additionally, expert is good to choose a leader of team. The leader is responsible, e.g., coordinating the work of teams. The leader should be someone who has experience in teamwork to increase the probability of achieving purpose.

- Stage 2.3. Identification of an Incompatibility Source

The identification of an incompatibility source is an indication of the place in which this incompatibility occurred. In the proposed approach, this stage is performed by brainstorming (BM) realized by a team of experts. It is possible to identify all sources of incompatibility. However, for a large number of incompatibilities, it is possible to use the Pareto rule (20/80), as is shown [21,23].

- Stage 2.4. Identification of Initial Causes

This stage relies on searching for an answer to the question: “What was happed that incompatibility occurred?” It is realized for each source (place occurrence of incompatibility) selected in stage 3 of the model. In this approach, it is proposed to use brainstorming (BM) and Ishikawa diagram (cause and effect diagram). The choice of these techniques resulted from, for example, its possible use to identify as many as possible causes and from the visualization groups of these causes [16,17,18,19]. This stage is realized in four steps.

◦ Step 2.4.1. Creating a List of Potential (Initial) Causes

In the team of experts, brainstorming (BM) is performed. The purpose is to identify the initial causes of incompatibility according to the source. According to the BM method, the leader (expert) notes all causes in a place visible to the team, e.g., table. After about 30 min, it is necessary to end the BM. As a result, a list with a large number of initial (potential) causes of incompatibility is obtained.

◦ Step 2.4.2. Delete not Real Submissions

From the list of potential (initial) causes of incompatibility, it is possible to delete not real submissions (causes). In this order, the leader (expert) verifies all causes. Then, delete from this not possible (not probability) causes of analyzed incompatibility. Based on the reduced list, the remaining steps of the model are performed.

◦ Step 2.4.3. Determine Thematic (Category) Groups of Causes

To systematically analyze, it is necessary to determine the so-called thematic groups of causes. In the proposed approach, the cause-and-effect diagram is used. Following the authors of the work [11,32], it is assumed that thematic groups can result from Rule 5M + E, that is, man, method, machine, material, measure, management, and environment. Moreover, it is possible to include categories, for example, environment, system, personnel, and suppliers.

◦ Step 2.4.4. Grouping All Causes into Categories

In this step, the team of experts groups the initial causes according to the categories assumed in step 4.3. of the model. To this aim, it is proposed to use the Ishikawa diagram (herringbone diagram, or fishbone diagram) by the method of Refs. [19,22,23]. Visualization is useful for better understanding the problem before the next action.

- Stage 2.5. Identification of Main Causes

The fifth stage of the model is the identification of the main causes of occurrence incompatibilities in the analyzed source. This process is realized as the second step of brainstorming (BM). It is possible to use other popular methods, e.g., Suzuki method (ABCD) [40]. It consists of performing brainstorming (BM) among a team of experts. The team of experts analyzes all potential (initial) causes determined in Stage 2.4. At the result, the team indicates the main cause, so the cause that has the greatest impact on the occurrence of incompatibility.

- Stage 2.6. Determining Improvement Actions

After the identified source and main causes of incompatibility, it is possible to determine improvement actions. Therefore, the purpose related to the main causes should be determined. Then, it is proposed to use the 5Why method. It concerns the search for an answer to the question of how to solve the problem, as is shown in [11,32].

The test of the proposed model is shown in the next part of the article.

## 3. Test of the Model

### 3.1. Material

**Figure 3:**
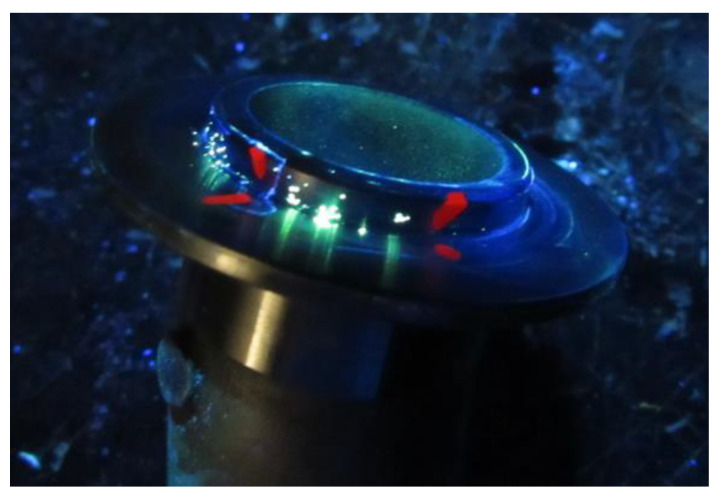
Porosity cluster on mechanical seal from alloy 410 identified by nondestructive testing (fluorescent method).

The test of the proposed model was realized for the defect of the material, i.e., porosity. This defect was frequently identified in the Polish industry. The product in which porosity was identified is the mechanical seal from alloy 410, which is a new-generation seal. The most often identified was the microporosity cluster (i.e., invisible to the naked eye), which was usually evenly spaced over the sealer surface. These formed chains (strands) or larger clusters of small cavities. These were formed mainly during the solidification of local volumes, which were isolated in the solidification process from the liquid metal supply. It is an undesirable phenomenon and is considered a product defect. A selected example of this incompatibility identified by the fluorescent method (FPI) on the mechanical seal is shown in Figure 3.

The mechanical seal is the casting and welded product. This product allows fluid retention (e.g., as a pump or mixer). One of them is mounted and sprung to accommodate possible shaft deflections or movements during bearing tolerances and nonvertical alignment due to manufacturing tolerances. The advantages of using mechanical seals include reduction of leaks, minimization of shaft or pump sleeve damage, self-adjustment of disc wear, minimization of bearing contamination, protection against corrosion of other devices, and possibility of vacuum sealing [11,41]. In turn, alloy 410 is stainless steel, which is martensitic in nature and is often used in a hardened state. It is used to ensure high strength and moderate resistance to heat and corrosion. The properties of alloy 410 are shown in Table 2, Table 3, Table 4 and Table 5.

The choice of this product resulted from incompatibility, which was often identified by nondestructive testing (NDT). This incompatibility was the porosity cluster (i.e., the occurrence of numerous small gas bubbles localized in a group with a geometric distribution) [44]. The control was carried out in a production service enterprise localized in Poland. The most frequently identified porosity clusters of the mechanical seal of alloy 410 were not acceptable [45,46]. These defects were not acceptable according to the PN-EN ISO 5817:2014-05 [47] and PN-EN 1090-2:2018-09 [48], e.g., the maximum sum of the cavity was more than 2% of the size cavity, or size of porosity cluster was more than 2 mm of the size of the cavity. Identified incompatibility was considered necessary to analyze to identify the sources of this problem. The porosity cluster has a negative impact on the strength and tightness of the product. In the case of mechanical seals, whose main task is to work under heavy loads, this defect is a disqualifying the seal for use. Hence, it was recognized to analyze this problem by using the proposed universal model.

### 3.2. Method

The controls were carried out with nondestructive testing (fluorescent method). Initially, the mechanical seal is chilled to a maximum temperature of 40 °C. Then, the mechanical seal is degreased and dipped in penetrant HM-406. The procedure of dipping in penetrant HM-406 is realized according to the instruction of this substance (see, e.g., [45]) and the experience of the quality control manager. The time of the dipping of the mechanical seal in the penetrant is about 10 min (and sometimes 5 min is enough). In the case of initial identification of incompatibility, this time is equal to 30 min (eventually longer). In the research carried out, the time to dip into the penetrant is equal to about 30 min because the porosity cluster was initially identified. Subsequently, the mechanical seal was left to drain the penetrant from the surface into the penetrant reservoir. The mechanical seal was then cleaned with water directly at a minimum distance of 300 mm under light UV. Cleaning was carried out with a water jet at room temperature (10–38 °C). According to [45], it was avoided to rinse out penetrant residues from the area of incompatibility space (maximum pressure 0.276 MPa). The time to rinse (clean) is 10 min. The mechanical seal is then dried in a drying oven (maximum temperature 70 °C). Developer ZP-4B in powder form were put on the dried product under air pressure (max. 0.172 MPa, time 10 min). Then, by compressed air (maximum pressure 0.034 MPa), the excess developer is related. The mechanical seal was checked in the control cabin under a UV lamp with minimum radiation intensity turned on (space 1200 μW/cm^2^, where maximum light intensity on the surface of the cabin was 20 lx). A TAM 146040 master plate was used to control the mechanical seal. After the control, the sealant was washed with water to remove the test substances.

- Stage 3.1. Determining the Main Incompatibility and Purpose of Analyse

The purpose was to improve the quality of the mechanical seal of alloy 410, by determining in a team of experts: the source of incompatibility, initial (potential) causes, main causes, and improvement actions.

- Stage 3.2. Choice of Team Experts

In the second stage, the team of experts was selected to assign responsibilities to improve the quality of the mechanical seal. For that, the method of choice team of experts was used, as is shown in three steps.

◦ Step 3.2.1. Choice of Number n Experts

In the first step, the entity selected two experts, an employee performing the production of the mechanical seal and an employee performing NDT inspections. These employees were chosen because of their knowledge, experience, and competencies in the context of the problem studied.

◦ Step 3.2.2. Calculation of the Number of Experts with a Team of Experts

In the second step, it was assumed that each of the two selected experts identified five consecutive experts among themselves. After using Formula (2), the required number of experts in the team was estimated (7):

(7) $$N=\frac{5^{2}\left(5-1\right)}{\left(5^{2}-9\right)+1}=\frac{100}{17}=5.88\approx 6,$$

where: N—number of experts required.

Each of the two selected experts pointed to the five next experts, where one expert was pointed twice. Therefore, it was estimated that the initial required number of experts should be equal to six experts. The group of initially selected experts included employees of the selected enterprise, i.e., the quality control manager ordering a need to solve the problem of porosity clusters on the mechanical seal, the employee conducting fluorescence testing and the NDT test manager, an employee working in production of mechanical seals, and authors of the article.

◦ Step 3.2.3. Determining Expert Competence Factor

According to the third stage, each of the initially selected experts determined their competence to analyze the porosity cluster on the mechanical seal. To this aim, Formulas (3)–(6) were used. The result is shown in Formula (8):

(8) $$\begin{array}{cc} K_{k1}=\frac{\left(9\times 0.1\right)+\left(0.50+0.35+0.20\right)}{2}=0.97 & K_{k4}=\frac{\left(8\times 0.1\right)+\left(0.20+0.15+0.10\right)}{2}=0.62\\ K_{k2}=\frac{\left(8\times 0.1\right)+\left(0.50+0.35+0.20\right)}{2}=0.92 & K_{k5}=\frac{\left(7\times 0.1\right)+\left(0.20+0.17+0.14\right)}{2}=0.61\\ K_{k3}=\frac{\left(8\times 0.1\right)+\left(0.20+0.15+0.10\right)}{2}=0.62 & K_{k6}=\frac{\left(7\times 0.1\right)+\left(0.20+0.17+0.14\right)}{2}=0.61 \end{array}$$

where: *K_k_*—competency factor for the i-th expert.

According to assumptions about the threshold value of the *K_k_* factor equal to 0.60, it was shown that all experts (T = N = 6) are competent to analyze the problem of the porosity cluster in the mechanical seal of alloy 410. Of these experts, the entity that used the method selected a leader, who was the quality control manager for NDT tests.

- Stage 3.3. Identification of Incompatibility Sources

In the third stage of the model, the source of incompatibility of porosity cluster on the mechanical seal was identified. During brainstorming (BM) conducted by the team of experts, it was concluded that the source of the porosity cluster concerned dissolved gases in liquid metal with components in liquid. In the case of lower metal temperatures, some of the dissolved gases separate from the solution. It is possible to trap them in the metal when it solidifies. Additionally, dissolved oxides frequently often make reactions with coal, and there are insoluble blisters in liquid and solidified metals. Hence, it was determined by a team of experts that porosity occurs during gas evolution in the metal when it solidifies. The confirmation of the right identified source on the porosity is literature of the subject, e.g., [49].

- Stage 3.4. Identification of Initial Causes

This stage relies on searching for an answer to the question: “What caused incompatibility to occur?” This stage was realized for the source of incompatibility (determined in the third stage). In this aim, the brainstorming (BM) and causes and effects diagram were used. This process was carried out in four steps.

◦ Step 3.4.1. Creating a List of Potential (Initial) Causes

In the first step, brainstorming (BM) was carried out among the team of experts. The purpose was to identify as many initial causes of porosity focusing on the mechanical seal as possible as a result of gas evolution from the metal as it solidifies. The list with initial causes was developed, and these causes were as follows.

1. significant nitrogen and hydrogen content in the arc atmosphere;
2. too high of a clotting rate;
3. metallurgical reactions concerning the formation of reaction products in gaseous form;
4. casting structure (problems with ensuring directional solidification);
5. interaction of iron oxide with carbon, which releases carbon monoxide and carbon dioxide;
6. possibility of moisture in the flux, e.g., during automatic welding;
7. rust on wire;
8. inadequate gas shield;
9. electrode moisture;
10. dirty casting mold;
11. employee mistakes;
12. little experience of the employee (resulting from a short period of work);
13. fatigue;
14. stress;
15. rush;
16. distraction;
17. lack of periodic training;
18. lack of TMP (Total Productive Maintenance);
19. lack of actual procedures and instructions;
20. inadequate number of controls during production;
21. impurities in sand;
22. water in the molding sand;
23. nuisance working conditions causing distraction, e.g., noise.

The brainstorming (BM) ended after about 30 min. As a result, 23 initial (potential) causes of the porosity cluster on the mechanical seal of alloy 410 were determined, the source of which was determined by the evolution of gases from the metal at the time of solidification. Furthermore, the identified causes were confirmed by other authors, who verified the causes of the porosity cluster, [14,16,27,50].

◦ Step 3.4.2. Delete Fake Submissions

In this step, to delete fake submissions, the leader (quality control manager with NDT) verified all the potential (initial) causes generated by the porosity cluster in the mechanical seal. After verifying all causes from the list, a single submission was deleted (i.e., 10—dirty casting mold), which was considered as not real. Additionally, submissions from 13 to 16 were combined as a single cause, i.e., a psychophysical state. As a result, a list with 19 initial (potential) causes was achieved.

◦ Step 3.4.3. Determine Thematic (Category) Groups of Causes

In the third step, a team of experts determined thematic (category) groups of the causes of porosity cluster on the mechanical seal of alloy 410. According to the proposed approach, the popular and often used categories (formula 5M + E) were selected, that is, man, method, material, machine, management, measure, and environment. These categories allow an effective and standardized way to group all generated causes of analyzed incompatibility.

◦ Step 3.4.4. Grouping all Causes into Categories

**Figure 4:**
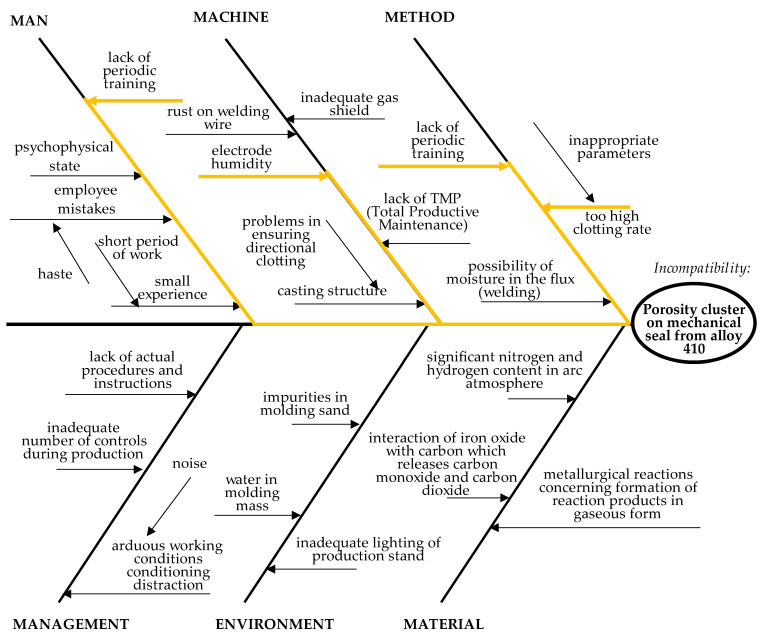
Ishikawa diagram for problem of porosity cluster on mechanical seal of alloy 410.

According to the fourth step, the team of experts has grouped all causes of the porosity cluster (from the list of causes determined in Step 3.4.2.) into categories (determined in Step 3.4.3.). As proposed, causes were grouped into categories in the Ishikawa diagram (Figure 4). After developing the Ishikawa diagram, it was assumed to determine the main cause of the porosity cluster on the mechanical seal.

- Stage 3.5. Identification of the Main Causes

In the fifth stage, the main causes of the porosity cluster in the analyzed source were identified. With this aim, the second stage of brainstorming was carried out among the team of experts. As a result, the team of experts concluded that the main causes were:

- too high of a clotting rate (inadequate parameters);
- lack of periodic training;
- electrode moisture.

The main causes were also noted in the Ishikawa diagram (Figure 4). To reduce or eliminate the main causes, improvement actions were determined.

- Stage 3.6. Determining Improvement Actions

**Figure 5:**
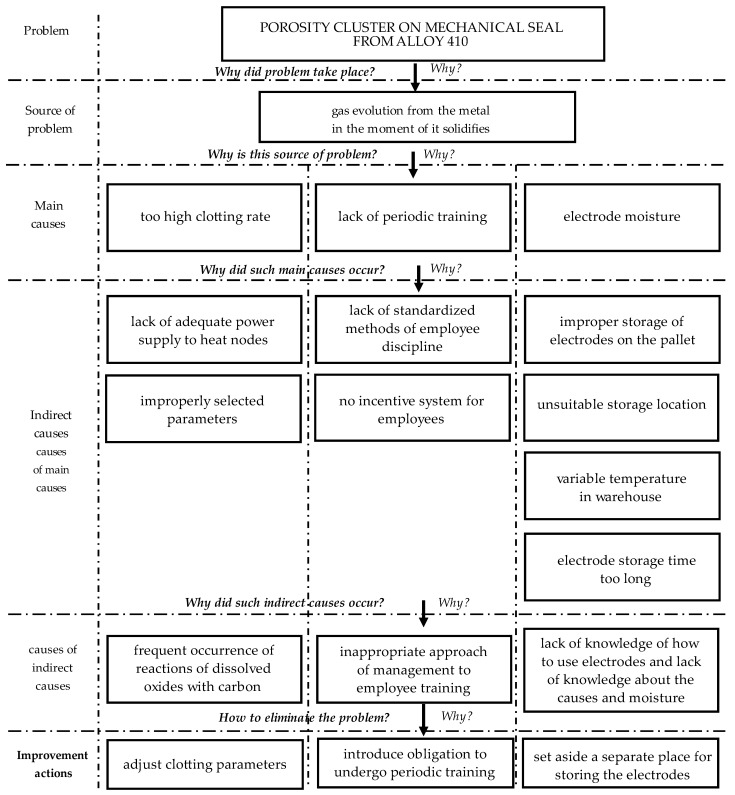
Analysis of 5Why method to determine improvement actions to reduce porosity cluster on mechanical seal of alloy 410.

At this stage, the team of experts is looking for an answer to the question “How to solve the problem with porosity clusters with mechanical seal of alloy 410?”. In the proposed approach, the 5Why method was used for that. The results are shown in Figure 5.

After analysis by the 5Why method, it was shown that the appropriate improvement actions are to adjust the clotting parameters, introduce the obligation to undergo periodic training, and set aside a separate place for storing the electrodes. According to the team of experts, these improvement actions will allow reducing the number of incompatibilities (porosity clusters) in the mechanical seal of alloy 410.

## 4. Discussion

Improving product quality is a key action of each company [51,52,53]. Despite the continuous development of enterprise actions [54,55,56], the search continues for effective instruments that support this process [2,15,57]. Therefore, the purpose was to develop a universal model supporting the improvement of product quality by coherent and repeatable determining causes of incompatibilities and actions that allow its elimination, where this model can be integrated with any research of product quality control. The model was tested for the incompatibility of the casting product, i.e., porosity cluster with the mechanical seal of alloy 410. These incompatibilities were identified by nondestructive testing (fluorescent method) in a Polish company. Initially, the objective was determined by using the SMART(-ER) method. In the analysis case, the aim was to improve the quality of the mechanical seal of alloy 410, by determining by a team of experts: source of incompatibility, initial (potential) causes, main causes, and improvement actions. Then, according to the method of choice assumed by the team of experts, it was selected experts who are assigned the responsibility to improve the quality of the mechanical seal. The group of selected experts included employees of the selected enterprise, the quality control manager ordering a need to solve the problem of porosity cluster on mechanical seals, the employee conducting fluorescence testing and the NDT test manager, an employee working in production of mechanical seals, and authors of the article. In the third stage of the model, the source of incompatibility was determined. For this, brainstorming (BM) was used, after which the team of experts concluded that a porosity cluster was formed during the evolution of the gases from the metal as it solidified. In the next stage, the initial (potential) causes were identified. At this stage, brainstorming (BM) was used again, after which 23 causes were initially determined. The leader of this team verified all causes, after which he considered that it is adequate to analyze 19 causes (potential) from all reported. These causes were grouped and visualized on the Ishikawa diagram according to rule 5M + E, that is, man, method, machine, material, measure, management, and environment. Then, the main causes of the porosity cluster in the mechanical seal were identified. As part of the second stage of brainstorming (BM), the team of experts concluded that the main causes were too high clotting rate (inadequate parameters), lack of periodic training, and electrode moisture. In the last stage, improvement actions were determined. It consisted of using the 5Why method. After analysis by the 5Why method, it was shown that the appropriate improvement actions are to adjust the clotting parameters, introduce the obligation to undergo periodic training, and set aside a separate place for storing the electrodes. According to the team of experts, these improvement actions will allow reducing the number of incompatibilities (porosity clusters) in the mechanical seal of alloy 410.

After test of the model, it was found that it is possible to combine the selected quality management tools into a coherent model, whose main aim is to identify the main causes of incompatibility and improvement actions, where this model will be universal, i.e., allow for the verification of any kind of incompatibility of the products, which were detected by any quality control methods.

Main benefits of the proposed model:

- assurance to determine causes of incompatibility in a coherent and repetitive way;
- simultaneously include the impact of the main causes on the problem occurs and determine adequate improvement actions;
- the possibility to implement this model to process identified problems (incompatibilities) identified by any method, for example, popular nondestructive testing or destructive testing;
- increase the profit of enterprise by making adequate improvement actions;
- assurance to enterprises of a model to thoughtful and accurate decision-making;
- analysis of possible incompatibilities among the properly selected team of experts, which allows for more accurate decision-making in the area of product improvement;
- categorize causes and their visualization in a noncomplicated way (standardizing the process of identifying source causes and improvement actions).

Another general benefit of the proposed model as part of the analysis of incompatibility identified by the NDT research:

- possibility to integrate the model with any product quality control, e.g., fluorescent method (FPI, i.e., nondestructive testing);
- use the model to analyze any industrial products;
- effectiveness in determining the source, initial causes, and main causes of incompatibility identified by the fluorescent test (FPI);
- the effectiveness of the NDT research in determining improvement actions for incompatibility was detected by the NDT research (e.g., fluorescent method—FPI).

In turn, some limitations of the proposed model are a selected team of experts to solve the problem, which is based on their self-esteem about their qualifications. In this case, experts must assess their own competence in an honest and truthful manner. In addition, improvement actions can be different in turn from enterprise resources. Additionally, it is important to mention that the proposed method ignores the statistical dispersion of quality parameters.

Therefore, future research will focus on creating the computer software supporting the proposed model. In addition, a dynamic decision platform is planned to be developed to make decisions about different kinds of incompatibility. This platform is planned to be created as part of information about different incompatibilities that occur with different types of products. Furthermore, as part of future research, it was assumed to compare the effectiveness of different combinations of improvement actions.

## 5. Conclusions

Ensuring the repeatability of identifying the causes of product incompatibility and determining improvement actions remains a challenge. In particular, it resulted in industry development (Industry 4.0) and future factories. In this area, it is useful to use different quality management tools. These instruments are effective in determining the causes of incompatibilities and improvement actions, which allow for eliminating these incompatibilities. However, if used inappropriately, they lose their effectiveness. Especially if these tools are used separately. Therefore, the aim was to develop a universal model supporting the improvement of product quality by coherent and repeatable determining causes of incompatibilities and actions that allow its elimination, where this model can be integrated with any research of product quality control. Mentioned combined tools were the SMART(-ER) method, the method of choice team of experts, brainstorming (BM), causes and effects diagram (Ishikawa diagram), and the 5Why method. The model was tested for the incompatibility of the casting product, i.e., porosity cluster with the mechanical seal of alloy 410. These incompatibilities were identified by nondestructive testing (fluorescent method) in a Polish company. As a result, it was shown that the main causes were too high of a clotting rate (inadequate parameters), lack of periodical training, and electrode moisture. Additionally, the appropriate improvement actions were to adjust the clotting parameters, introduce an obligation to undergo periodic training, and set aside a separate place for storing the electrodes.

The proposed combination of selected quality management tools in a coherent model allows for the identification of the main causes of incompatibility and choice of improvement actions. In addition, this model can be combined with any quality control. Additionally, this model is universal because the techniques used in this model have universal application. Hence, it can be used to allow us to verify any kind of incompatibilities of products, which were detected by any product quality controls. Therefore, it can be used by any type of company to improve the quality of the product. Future research will focus on implementing this model in computer software.

## Figures and Tables

**Table 1:** Values to determine expert competence factor. Own study based on [35].

Value of Points for Expert Self-Assessment			
Points	Description		
0–1	The expert does not know problem		
2–3	The expert hardly knows the problem, but it is within his area of interest		
4–6	The expert knows the problem to a satisfactory degree, but does not participate in its practical solution		
7–9	Experts know the problem well and participate in its practical solution		
10	The expert knows the problem perfectly well, where this problem belongs to a narrow specialization of expert		
Factor of arguments k_a_			
Source	Arguments		
	a_1_	a_2_	a_3_
Theoretical analysis carried out by expert	0.20	0.15	0.10
Expert’s practical experience	0.50	0.35	0.20
Knowledge of works by native authors	0.05	0.04	0.03
Knowledge of works by foreign authors	0.05	0.04	0.03
Expert intuition	0.20	0.17	0.14

**Table 2:** Properties of alloy 410—mechanical and physical. Own study based on [42,43].

Properties	21 °C	100 °C	500 °C	649 °C	788 °C
Thermal expansion coefficient [μm/m °C]	-	9.8	11.2	11.7	11.9
Thermal conductivity [kcal/°C]	-	21.4	24.7	-	-
Modulus of elasticity [×10^5^ MPa]	2	-	-	-	-

**Table 3:** Properties of alloy 410—tensile strength. Own study based on [42,43].

Tensile Strength, MPa	60–75
0.2% yield point [MPa]	32–42
Elongation [%]	20–40
Reduction of surface [%]	50–75

**Table 4:** Properties of alloy 410—tempering temperature. Own study based on [42,43].

Tempering Temperature [°C]	-	149	260	371	566	621	649	704	760	816
Tensile strength [MPa]	193.5	188.5	181.6	181.4	124.1	117.5	113	101.8	96.5	131.8
0.2% yield point	149.8	148.6	143.6	144.7	110.3	103.7	99.1	84.2	77.9	88.6
Elongation, %	17	17.3	16.8	16	20.8	21.3	22	23.5	25	19.5
Reduction of surface, %	56.8	59.7	61.1	61.1	67.2	66.1	66.5	68.8	69.9	59.6
Brinell hardness	388	388	361	361	255	235	229	207	189	257

**Table 5:** Chemical composition of alloy 410. Own study based on [42,43].

[%]	Cr	Mn	Ni	C	Si	P	S	Fe
Min.	11.5	-	-	0.08	-	-	-	-
Max.	13.5	1	0.75	0.15	1	0.04	0.03	Balance

## Data Availability

Not applicable.
